# Hybrid disease prediction approach leveraging digital twin and metaverse technologies for health consumer

**DOI:** 10.1186/s12911-024-02495-2

**Published:** 2024-04-05

**Authors:** Chaitanya Kulkarni, Aadam Quraishi, Mohan Raparthi, Mohammad Shabaz, Muhammad Attique Khan, Raj A. Varma, Ismail Keshta, Mukesh Soni, Haewon Byeon

**Affiliations:** 1 Department of Computer Engineering, Vidya Pratishthan’s Kamalnayan Bajaj Institute of Engineering and Technology, Baramati, Pune, 413133 Maharashtra India; 2 M.D. Research, Intervention Treatment Institute, Houston, TX USA; 3 Software Engineer, Alphabet Life Science, Dallas, TX 75063 USA; 4 Model Institute of Engineering and Technology, Jammu, J&K India; 5 Department of Computer Science and Mathematics, Lebanese American University, Beirut, Lebanon; 6 Symbiosis Law School (SLS), Symbiosis International (Deemed University) (SIU), Vimannagar, Pune, Maharashtra India; 7 Computer Science and Information Systems Department, College of Applied Sciences, AlMaarefa University, Riyadh, Saudi Arabia; 8 Dr D Y Patil Vidyapeeth, Dr. D. Y. Patil School of Science and Technology, Pune, 411033 India; 9 Department of Digital Anti-Aging Healthcare, Inje University, Gimhae, Republic of Korea 50834

**Keywords:** Disease prediction, Bioinformatics, Healthcare, Digital twin, Deep neural networks

## Abstract

Emerging from the convergence of digital twin technology and the metaverse, consumer health (MCH) is witnessing a transformative shift. The amalgamation of bioinformatics with healthcare Big Data has ushered in a new era of disease prediction models that harness comprehensive medical data, enabling the anticipation of illnesses even before the onset of symptoms. In this model, deep neural networks stand out because they improve accuracy remarkably by increasing network depth and making weight changes using gradient descent. Nonetheless, traditional methods face their own set of challenges, including the issues of gradient instability and slow training. In this case, the Broad Learning System (BLS) stands out as a good alternative. It gets around the problems with gradient descent and lets you quickly rebuild a model through incremental learning. One problem with BLS is that it has trouble extracting complex features from complex medical data. This makes it less useful in a wide range of healthcare situations. In response to these challenges, we introduce DAE-BLS, a novel hybrid model that marries Denoising AutoEncoder (DAE) noise reduction with the efficiency of BLS. This hybrid approach excels in robust feature extraction, particularly within the intricate and multifaceted world of medical data. Validation using diverse datasets yields impressive results, with accuracies reaching as high as 98.50%. DAE-BLS’s ability to rapidly adapt through incremental learning holds great promise for accurate and agile disease prediction, especially within the complex and dynamic healthcare scenarios of today.

## Introduction

In current era, society is experiencing a significant demographic shift toward an ageing population. Projections indicate that by 2050, the number of individuals over 60 will surpass 300 million [1]. A gradual increase in the prevalence of chronic diseases accompanies this ageing trend. The custom of planned hospital visits for re-examinations and treatments falls short when it comes to the long-term observation and treatment that the elderly and people with chronic diseases frequently require, which has a negative impact on patient well-being and mental health. Better patient outcomes now depend heavily on early disease prediction, and the concept of a digital twin offers a fresh perspective on this. In recent years, with the rapid development of Internet of Things technology, related applications have also appeared in the healthcare field, such as patient identity recognition, disease diagnosis, cloud data storage [2, 3], physiological signal detection [4, 5], etc. Setting up a remote community hospital that uses digital twin and metaverse technologies to improve these applications [6] can connect medical resources in the same area, creating a comprehensive pool of medical resources that eases the burden of not having enough medical resources. By linking related medical resources, a comprehensive pool of resources is created, which lessens the burden of insufficient medical resources. Tele Community Hospital uses digital twin technologies to assist patients in the grassroots community in resolving issues. The hospital is cantered around a team of experts and uses electronic medical data shared by various treatment points in the community. This model greatly strengthens the connection between community members and community hospitals, breaking the previous restrictions on the time and place of seeing a doctor. How to use medical data in community hospitals, enhanced by digital twin solutions, for fast and accurate disease prediction is a problem that needs to be solved.

Today, deep neural networks form the basis of most algorithm models used for disease prediction, and incorporating digital twin technology into this landscape promises transformative potential. This study [7] employed deep learning, bolstered by digital twin insights, to forecast retinal markers of cardiovascular disease. In order to detect myocardial infarction automatically, researchers [8], leveraging digital twin simulations, created a convolutional neural network. Using more straightforward automated screenings of MB-creatine kinase mass that employ monoclonal antibodies, physicians can identify myocardial infarction as early as two hours following coronary blockage. Myoglobin and troponin are two more potential indicators of cardiac necrosis. Healthfog is a system introduced in this paper [9], utilizing deep neural networks and digital twin concepts to detect cardiovascular illness.

Yet another difficulty with deep neural networks, as well as digital twin integration, is something known as “catastrophic forgetting” [10], which describes the fact that once certain parameters, such as the number of hidden layers, the number of neurons, the number of times, and the learning rate, have been confirmed when defining the structure of the neural network and its digital twin counterpart, they cannot be changed unless the network and digital twin system are reset. Every time new detection data is acquired, all of the data must be retrained, which is why it is critical to construct the network model and its digital twin framework in advance to ensure smooth integration and adaptation. Due to the shortcomings of existing deep learning techniques, a ground-breaking remedy known as the Broad Learning System (BLS) was created. When combined with digital twin technology, BLS expands the possibilities for innovation and disease prediction. Literature [11] has introduced the BLS model, renowned for its straightforward design and rapid processing capabilities. In this era of digital twins, BLS’s potential is magnified, allowing for versatile applications in healthcare [12].

The structural disparities between a BLS and a deep neural network become even more profound when examined in the context of time series forecasting and digital twin-enabled simulations [13]. BLS exhibits a preference for building networks in the direction of “width” rather than “depth,” a strategic choice that aligns with the principles of digital twin systems. The absence of hidden layers results in swift training times, eliminating the need for gradient descent in weight updates. Gradient instability and sluggish training are two problems that traditional approaches have to deal with. The Broad Learning System (BLS) sticks out as a good substitute in this situation. By using incremental learning, it circumvents the issues with gradient descent and enables rapid model reconstruction. BLS struggles to extract complicated features from complex medical data, which is one of its issues. Furthermore, the dynamic network topology of BLS, coupled with digital twin simulations, allows the model to evolve over time, enhancing its adaptability. It’s in this synergy of BLS and digital twin technology that incremental learning approaches [11], guided by principles of digital twin modeling [14], shine. This approach dictates that only the portions of the knowledge base that have changed due to new data need to be updated, eliminating the need to recreate the entire model each time new information surfaces.

However, a challenge arises when considering the integration of BLS and digital twin frameworks. In the original BLS model, the weights of the input and mapping layers are generated randomly, which, in the context of digital twin systems, necessitates a more purposeful approach to model initialization [15]. As it stands, this configuration isn’t an ideal match for the prediction of today’s complex, high-dimensional medical datasets in conjunction with digital twin data environments. Creating a predictive model that accommodates both the intricacies of complex medical data and digital twin simulations requires a novel strategy.

It takes a creative approach to build a predictive model that can handle the complexities of digital twin simulations and sophisticated medical data. The authors of this work suggest a novel approach to address this problem: an incremental prediction model based on width learning and denoising autoencoders (DAE-BLS). With digital twin technology serving as the backdrop, this hybrid approach overcomes the hurdles of extended training times and the complexities of model updates while enhancing adaptability to the dynamic and intricate data environments. The digital equivalent of a physical item or gadget is called a digital twin. An IoT application can be successfully deployed and used with the aid of a digital twin. Another name for a digital twin is a twin or a shadow.

The DAE-BLS model, in conjunction with digital twin technology, offers rapid real-time performance, combining the benefits of incremental learning and the feature extraction capabilities of denoising autoencoders, which are particularly well-suited for the turbulent digital twin landscapes. This synergy not only ensures the accurate and agile prediction of diseases but also bridges the gap between digital twin technology and advanced healthcare solutions, pushing the boundaries of what is possible in this digital age.

## Related work

Disease prediction refers to the use of collected medical data, such as the patient’s social and economic status, clinical information, and physiological signals, to construct an algorithm model to determine whether the patient will develop a disease in the future. Disease prediction is the process of building an algorithm model to predict a patient’s likelihood of contracting an illness in the future using data that has been gathered from medical records, including the patient’s social and economic position, clinical information, and physiological signals. However, how to extract robust characteristics from chaotic and complex data is a challenge because of the intricacy of the medical data itself. Many researchers have considered using unsupervised algorithms for feature extraction. In this respect, autoencoders are Very extensive research has been done.

This study [16] proposed a denoising autoencoder (DAE) with the belief that a decent feature extraction should be able to capture the stable structure of the input signal and have some robustness. The main way that DAE differs from a traditional autoencoder is that it adds noise to the input and then uses the damaged, noise-filled version of the sample to recreate the original, noise-free input. DAE’s learned features so gain from this training approach as well. Replicating the characteristics of the original input data accurately makes it useful in situations when the data is unpredictable. As a result, DAE’s learnt features benefit from this method of training as well. Useful in contexts with erratic data, as it faithfully reproduces the features of the original input data. The next step in disease prediction after feature extraction is to develop an appropriate algorithm model. When a hereditary illness is passed down from one generation to the next, its signs and symptoms often worsen and manifest earlier in life. We refer to this phenomenon as anticipation. Neural network model with bidirectional recurrent architecture for illness risk prediction. They selected three chronic diseases with high incidence rates for the purpose of predicting each person’s risk for each disease. Methods for disease prediction and evaluation using deep neural networks have been proposed in the medical profession, however there are still many obstacles to overcome because of the complexity of medical data.

Autoencoder is a well-liked unsupervised feature extraction method in machine learning. An input layer, an output layer (used for decoding), and a hidden layer (used for encoding) make up a neural network. The objective of this network is to reconstruct the input in order to enable the hidden layer to fully comprehend the prominent features of the input. Information is transferred from the input layer to the output layer during the forward propagation process, when each neuron’s output is calculated successively, layer by layer. Backpropagation is the technique of updating each neuron’s weight in a neural network, working backwards from the output layer towards the input layer. Inspired by the paper released in [17] and further developed in [18–20], a stack of shallow autoencoder models first encodes the input data and then reconstructs it to learn its features. This article [21] presented a deep network termed a convolutional autoencoder to extract features from complicated image data. In order to improve the autoencoder’s node, researchers implemented a convolutional layer and a pooling layer. The automatic encoder is convolutionally trained and produces superior results when fed picture data directly. To improve the overall progress of treatment plans and processes, new techniques are adapted [22, 23]. Cancer digital twins are a relatively new approach that uses a range of AI and biological methodologies to process input data and then represents exact therapy procedures [24]. Since ML methods yield legitimate and trustworthy conclusions without requiring deep learning about the input data, they are regarded as important analytical techniques [25]. AI models are taught to gradually assimilate new information through the use of the incremental technique in machine learning. This gives models the capacity to maintain and advance existing knowledge, providing a basis for further development. In machine learning, autoencoder is a popular unsupervised feature extraction technique [26, 27]. Because of this, machine learning (ML) is being used more and more in a variety of sectors, such as engineering, economics, and healthcare [28]. Reconstructing the input is the aim of this network to allow the hidden layer to completely understand the salient characteristics of the input. The transformation of raw data picture files, for instance, into numerical characteristics that may be utilised with machine learning methods, is a typical use case for feature extraction [29].

### Predictive model based on deep neural network

Literature [30] made a bidirectional recurrent neural network model for predicting disease risk. For individual disease risk prediction, they chose three long-term diseases with high rates of occurrence. In this study, the design of a model to predict the chance of disease was looked at. Introduced a multimodal learning strategy, which involves using multimodal learning on different types of medical data and fusing the traits that were learned. The phenomenon referred to as “catastrophic forgetting” presents an additional challenge to deep neural networks and digital twin integration. This refers to the inability to modify specific parameters, such as the number of neurons, hidden layers, number of times, and learning rate, once they have been confirmed during the definition of the neural networks and its digital twin’s structure, unless the network and digital twin system are reset. This strategy is used to predict the risk of disease. Researchers merged support vector machines and deep belief networks in the literature [31].

Based on the research that has already been done, there have been many great results in the study of disease prediction models, but there are still some problems:

1. Even though the deep neural network has gotten pretty good at making predictions, the way it is trained makes it hard to train and often takes a long time. At the same time, medical data in the real world is growing all the time. Existing prediction models can’t keep the knowledge they’ve learned from training, so they have to retrain every time the data changes. A concept of a bidirectional recurrent neural network for illness risk prediction. They selected three chronic diseases with high incidence rates for the purpose of predicting each person’s risk for each disease. A model to forecast the likelihood of an illness was designed. Introduced a multimodal learning technique that combines the learnt features with multimodal learning applied to various medical data types.
2. The BLS model can be trained quickly, and the model can be quickly reconstructed through incremental learning. However, its basic structure is not good at learning features, and it is not good for environments with a lot of complex data.

This paper suggests a prediction model based on denoising autoencoder and width learning (DAE-BLS) to solve the problems listed above. Next, the details of how it was built are given.

## Denoising autoencoders with wide learning (DAE-BLS)-based predictive models

**Fig. 1:**
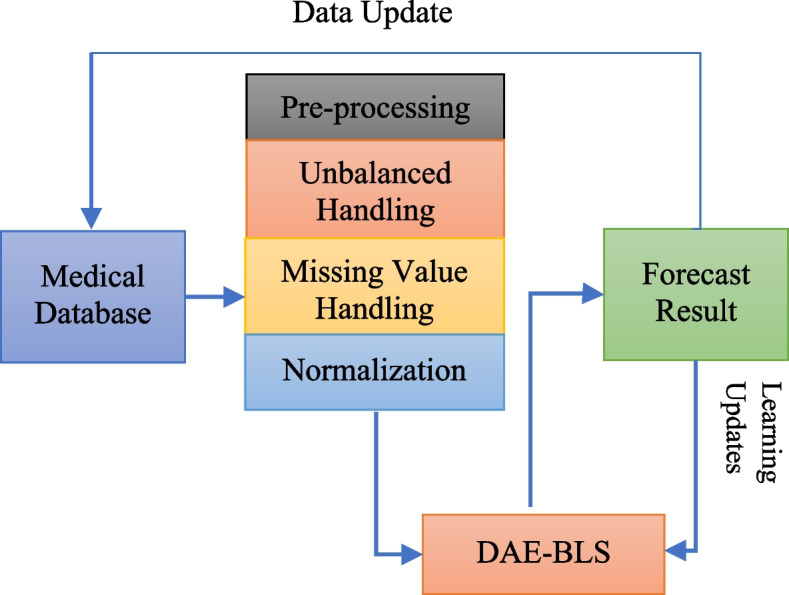
Disease prediction model structure

This section will explain how the model suggested in this paper will be put into place. Figure 1 shows how the model is put together. The model in this paper can be generally split into two parts: the pre-processing module and the DAE-BLS module. When time series predicting and digital twin-enabled simulation are considered, the structural differences between a BLS and a deep neural network become even more pronounced. It may be observed that BLS favors network construction along “width” rather “depth,” a deliberate decision that is consistent with the concepts of digital twin systems. The model is used by medical equipment that you wear. The data from the electronic medical records are used as input. Using visual, aural, reading, writing, and kinaesthetic approaches to teach a subject is known as multimodal learning. By aligning the delivery of knowledge with the most effective way for students to learn, it aims to raise instructive standards. This model’s benefits are that it is easy to use and works well. It also adapts well to an unstable environment, so the results it predicts can be used in time to make good decisions.

### Data pre-processing

Due to noise, incompleteness and inconsistency, the actual collected medical data cannot be directly used for prediction tasks. Therefore, data pre-processing steps are required before data analysis to extract effective data features. Data pre-processing including missing value processing, data standardization processing, data imbalance processing, etc. The pre-processing process is shown in Algorithm 1.

**Figure:**
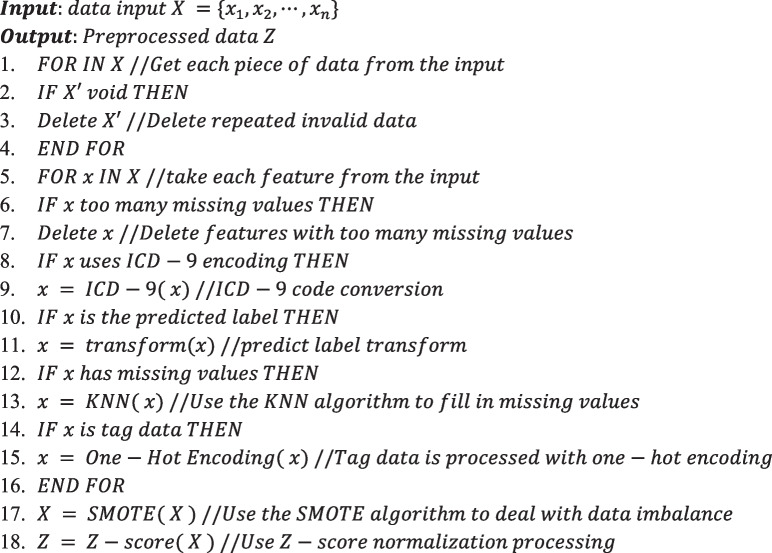
**Algorithm 1** Pre-processing process

There are many reasons why there are missing values in the medical data that has been gathered, such as patients wanting to protect their privacy, nurses making mistakes, patients not getting a full physical exam, etc. When missing data is used directly for classification, it will lower the rate of accuracy. So, for the information This study uses a better way to explain the k-nearest neighbour (KNN) algorithm to fill in missing values for the missing problem. The sample has been used up.Most of the time, different evaluation factors in medical data have different sizes and units, which will change the results of analysing the data. This paper decides to do this in order to get rid of the dimensional influence between indicators and to take into account the effect of more medical data on parameter definitions. The findings of data analysis are often affected by the size and unit differences between various assessments elements found in medical data. In order to eliminate the dimensional influence between indicators and account for the impact of additional medical data on parameter definitions, this study makes this decision. When compared to the heart failure dataset, the training time improvements in the diabetic patient datasets are less pronounced for both the original BLS and the improved DAE-BLS. There is a need for more studies with bigger sample sizes and more complicated databases, as this discrepancy may be related to variables like dataset size and complexity. For normalisation, use the Z-score normalisation method. This method gives the mean and standard deviation of the original data to standardise the data so that the processing data fits the standard normal distribution. This means that the mean is 0 and the standard deviation is 1, and the transformation function for the mean is the same as the transformation function for the standard deviation.

1 $${X}^{*}=\frac{x-\mu }{\sigma }$$

Among the variables considered, µ represents the average value of the entire set of sample data, while σ denotes the standard deviation of the samples. The distribution of disease data is characterised by an evident imbalance, with a much smaller number of categories representing diseased instances compared to the number of categories representing normal instances. In order to address the issue of data imbalance, the present study employs the Synthetic Minority Over-sampling Technique (SMOTE) Algorithm as a pre-processing method. The method under consideration exhibits dissimilarities when compared to the oversampling technique that relies on the straightforward duplication of minority class data. The proposed method generates additional instances of the minority class by employing linear interpolation techniques on the current minority class samples.

### DAE-BLS model

1. Model structure

**Fig. 2:**
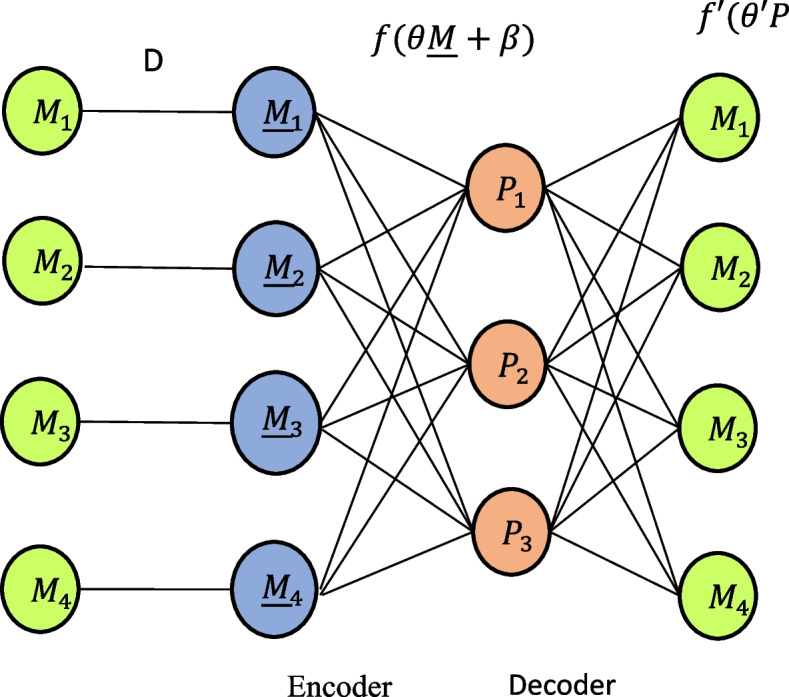
DAE structure

DAE is an effective variant of autoencoder. Its main idea is: first manually add random noise to the original input data to make it a corrupted version; then train the autoencoder so that it can be reconstructed from the corrupted version of the data the original input. The DAE-BLS model uses the patient’s data to forecast the likelihood that the patient will get unwell in the future, following the pre-processing module. Additionally, the model’s flexibility is increased by using the recently created data to update the model’s parameters gradually. The DAE structure is shown in Fig. 2.

**Fig. 3:**
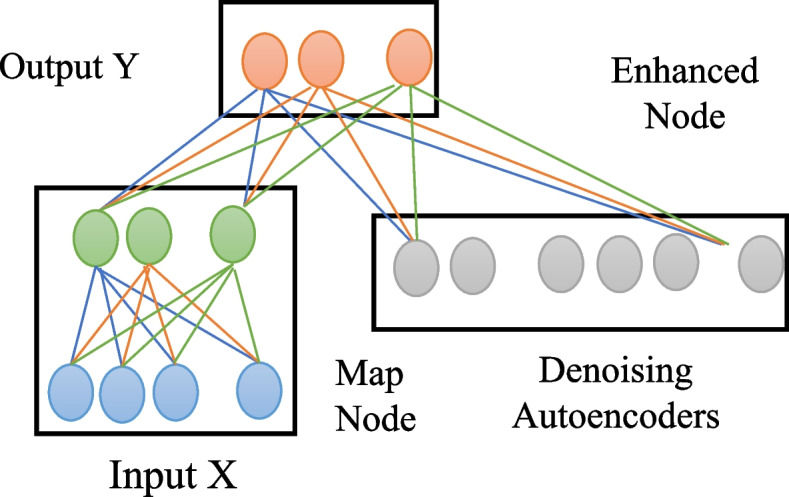
DAE-BLS model structure

In Fig. 3, D refers to adding random noise to the original input $M$ to make it a corrupted version $\underline M$, then let P$=f(\theta \underline M+\beta )$. Among them, $\theta$ is a mapping matrix to be learned, and β is a deviation vector, the obtained P is the hidden layer obtained after encoding, which is the feature of the original data that is expected to be extracted. The decoder part relies on the feature representation P to reconstruct the original data $\underline M$, and optimizes the self-encoder by minimizing the construction error. Parameters. The features extracted from the noise-polluted samples are more representative of the essence of the original input and more adaptable to noisy environments. A common use case for feature extraction is the conversion of unprocessed data image files, for example into numerical features that may be used with machine learning techniques. By extracting the geometry of an object or the redness value from an image, data scientists can develop new features that are appropriate for machine learning applications. BLS is simple and efficient, but it directly uses the original data for simple processing and then enters the system. It cannot extract enough features from complex and noisy medical data, resulting in low accuracy of the prediction model. For complex and chaotic medical data environment, in order to improve the feature extraction capability of BLS, this paper introduces DAE into the architecture design of BLS, thus proposing the DAE-BLS model, whose structure is shown in Fig. 3. The DAE-BLS model combines the characteristics of DAE and BLS, it not only ensures the efficient computing ability of the model, but also enhances the feature extraction ability of the model.

(2) Complexity analysis of the model

**Fig. 4:**
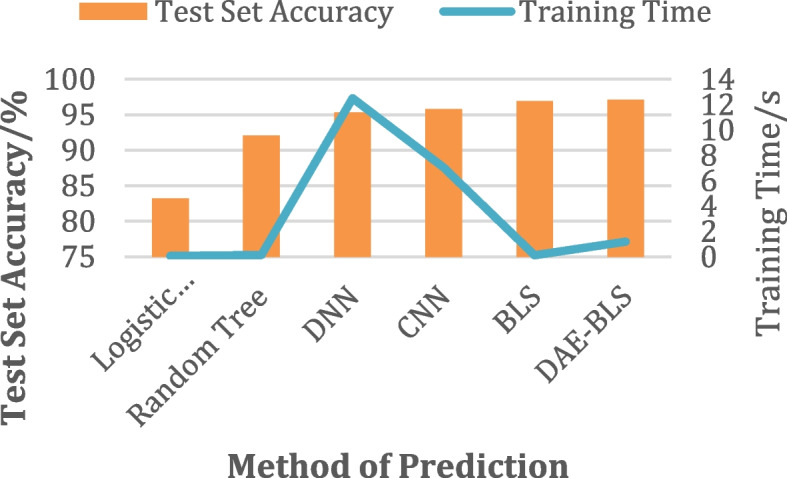
Comparisons of experimental results for heart failure

The training process for the majority of deep neural networks typically involves two distinct stages, namely forward propagation and reverse propagation. Deep neural network training necessitates considerable thought in order to stabilize learning, start with normalized input data. For efficient gradient propagation, use an appropriate activation function. Use weight initialization techniques to keep gradients from inflating or vanishing. Since the network structure of ordinary neural networks is frequently set and unchangeable throughout training, many models achieve the goal of increasing accuracy by repeatedly changing the network topology. Experts utilize the Convolutional Neural Network (CNN) machine learning method and K-Nearest Neighbor (KNN) machine learning algorithm to accurately predict diseases. A collection of disease symptoms is necessary for disease prediction. Backpropagation is a neural network technique that includes adjusting each neuron’s weight, starting from the output layer and working backwards towards the input layer. Using the loss function, this weight update is carried out layer by layer. This layer-by-layer gradient calculation is part of the process of updating each neuron’s weight. Hence, in scenarios where the network comprises numerous layers, the deep neural network is prone to encountering local optima and experiencing the vanishing gradient problem. Instances of gradient explosion, sluggish convergence, and similar phenomena are seen. When incremental learning is not employed, the training procedure of the DAE-BLS model follows a similar approach to that of typical neural networks. The training is conducted by specifying the amount of samples and network parameters. One of the examples, as illustrated in Fig. 4 The model’s hidden layer is comprised of two components, namely mapping nodes and enhancement nodes.

The DAE-BLS model uses the feature ${T}^{n}$ extracted by DAE as the mapping layer, ${T}^{n}=\left[{T}_{1},{T}_{2},\cdots ,{T}_{n}\right]$. Then the features obtained by the mapping layer are input into the enhancement layer part of the model, and ${H}_{j}$ is used to represent the j^th^ group of enhanced nodes, then there is

2 $${H}_{j}={\xi }_{j}\left({T}^{n}{W}_{hj}+{\beta }_{hj}\right),j=\text{1,2},\cdots ,m$$

Among them, ${\xi }_{j}$ is a nonlinear activation function; ${W}_{hj}$and ${\beta }_{hj}$ are random weights and biases respectively. Then, m groups of enhanced nodes are concatenated into ${H}^{m}=\left[{H}_{1}\cdot {H}_{2},\cdots ,{H}_{m}\right]$. Finally, the output of the enhanced layer and the mapping layer the outputs are spliced together, recorded as $A=\left[{T}^{n}\mid {H}^{m}\right]$, and the final output of the model is

3 $$Y=A{W}^{m}$$

Among them, ${W}^{m}$ is the weight from the hidden layer to the output layer. Since ${W}_{hj}$ and ${\beta }_{hj}$ in the enhancement layer mentioned above are randomly generated and remain unchanged during the training process, the parameters of the DAE as the mapping layer also remain unchanged, so the whole network needs to learn only ${W}^{m}$ weights, and ${W}^{m}$ can be quickly calculated by ridge regression. For ordinary neural networks, the weights are gradually updated using gradient descent one by one. For the width learning system, there is no weight update process, but the weights are calculated directly in one step.

In contrast to the deep neural network with several hidden layers, the DAEBLS model has a straightforward architecture, a reduced number of parameters requiring updates, and simplified weight computation. In contrast to the original BLS model, the model exhibits a DAE structure within the mapping layer, resulting in enhanced feature extraction capabilities. Furthermore, when compared to alternative deep neural network models, the model architecture presented in this study exhibits dynamic characteristics during the training procedure. The incremental technique in machine learning teaches AI models to gradually take in new information. This provides models with the ability to preserve and improve upon current knowledge, serving as a foundation for ongoing progress. Incremental learning has a number of benefits over classical machine learning, which necessitates a training set beforehand: (1) It can learn without a large enough training set before it starts operating; (2) It can continually learn to grow better while the system is operating; and (3) It can adjust to modifications in the target concept. This dynamism allows for efficient updates via incremental learning, hence enhancing the model’s training efficacy.

(3) Incremental learning method of the model

During the training process of ordinary neural networks, the network structure is often fixed and immutable, so many models achieve the purpose of improving accuracy by continuously modifying the network structure and then repeating training. Since the network topology of regular neural networks is frequently set and unchangeable throughout training, many models achieve the goal of increasing accuracy by repeatedly changing the network structure. The trained model dynamically grows the model structure, and the dynamic incremental learning algorithm may effectively utilize this to update the model’s weight in a convenient and effective way.

If the model cannot achieve the expected accuracy after training, the common solution is to insert additional enhanced nodes to obtain better performance. In order to ensure the performance of the model, this paper uses BLS incremental learning algorithm [11] based on Incremental learning algorithm for DAE-BLS method, the process is as follows.

- Step 1: Ensure that the model structure remains unchanged. After the enhancement nodes are added, the new hidden layer becomes

4 $${A}^{m+1}=\left[A\mid \xi \left({T}^{n}{W}_{m+1}+{\beta }_{m+1}\right)\right]$$

Among them, $\xi$ is the activation function; ${W}_{m+1}$ and ${\beta }_{m+1}$ are the new random weight and random bias of the enhancement layer, respectively.

- Step 2: Calculate the pseudo-inverse matrix of ${A}^{m+1}$, namely

5 $${\left({A}^{m+1}\right)}^{+}=\left[{A}^{+}-D{B}^{T} {B}^{T} \right]$$

In

$${B}^{T}=\{{C}^{+}, C\ne 0 {\left(1+{D}^{T}D\right)}^{-1}{B}^{T}{A}^{+}, C=0 , C=\xi \left({T}^{n}{W}_{m+1}+{\beta }_{m+1}\right)-AD, D={A}^{+}\xi \left({T}^{n}{W}_{m+1}+{\beta }_{m+1}\right).$$

- Step 3: Update the output weight ${W}^{m+1}$ as

6 $${W}^{m+1}=\left[{W}^{m}-D{B}^{T}Y {B}^{T}Y \right]$$

This method only needs to calculate the pseudo-inverse of the newly added enhanced node on the basis of the original hidden layer, instead of calculating the entire ${A}^{m+1}$, which greatly reduces the update time and makes it possible to dynamically update the output weight.

In summary, the training steps of the DAE-BLS model are shown in Algorithm 2.

**Figure:**
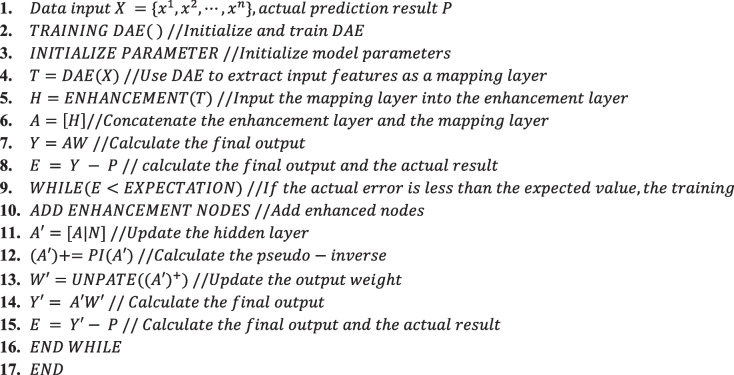
**Algorithm 2** DAE-BLS training steps

## Experimental detail and result analysis

In this paper, multiple simulation experiments have been carried out on data sets with different formats and different data volumes to verify the performance of the proposed model. This experiment is based on the Python language, Tensorflow and PyWavelets and other frameworks, and the hardware conditions are NVIDIA GeForce RTX 2070 SUPER GPU, AMD Ryzen 536,006-Core Processor 3.60 GHz CPU, 16GB memory.

### Structured data

This paper uses the Diabetes 130-US hospitals diabetes data set [16, 17] for simulation experiments. The diabetes data set collects 10 years (1999–2008) clinical care information of 130 hospitals and IDNs in the United States. The data set contains a total of 101,766 cases More than 50 characteristics of patients collected by the hospital, including patient number, race, gender, age, type of admission, length of stay, medical specialty of the admitting physician, number of laboratory tests, HbA1c test results, diagnosis, number of medications, diabetes medications The number of emergency visits in the year before hospitalization, etc.

This paper uses the model constructed to predict the patient’s readmission. The readmission labels in the data set are divided into three categories: readmission within 30 days, admission after 30 days, and no admission. Digital twins serve as the real-time digital replica of a physical system or process and are employed in system optimization and simulation. One method for creating a digital twin model using data is to use neural networks, particularly in cases where a physics-based model is unavailable or not very precise.

Another dataset is the dataset of hospitalized patients with heart failure [32], which uses electronic health data collected from patients hospitalized in a hospital. There are obvious differences in the system, so this data set is more suitable for medical system. There are multiple prediction labels in the data, and death within 28 days and readmission within 28 days are taken as the prediction labels.

#### Experimental results

1. Prediction of readmission for heart failure

After the above pre-processing, a heart failure data set containing 2516 pieces of 172-dimensional medical data was obtained. It was noticed that the data volume of this data set was relatively small, and a variety of algorithm experiments were compared on this data set. The results are shown in the Table 1. The result comparison is shown in Fig. 4.

**Table 1:** Heart failure prediction experiment results

Method	Training Accuracy	Test Accuracy	Training Time/s
Logistic Regression	85.25	83.24	0.07
Random Tree	96.95	92.10	0.14
Deep Neural Networks (DNNs)	98.95	95.34	12.51
Convolutional Neural Networks (CNNs)	99.44	95.82	7.05
Wide Neural Network (BLS)	98.79	96.92	0.13
DAE-BLS	99.55	97.12	1.20

It can be seen from the comparison that under the premise of ensuring the prediction accuracy, compared with the deep neural network, the neural network with a wide structure (BLS and DAE-BLS) not only achieves the best accuracy (96.92% and 97.12%), Moreover, the time required for training is greatly reduced, and the performance is effectively improved. Since the network topology of regular neural networks is frequently set and unchangeable throughout training, many models achieve the goal of increasing accuracy by repeatedly changing the network structure.

This is because the structure of the width neural network is simple and does not need to be solved by gradient descent. However, due to the limited size of the data set, there may be some deviations in the results, so Next, we will use the diabetes dataset with more data and more chaotic data to conduct experiments.

**Fig. 5:**
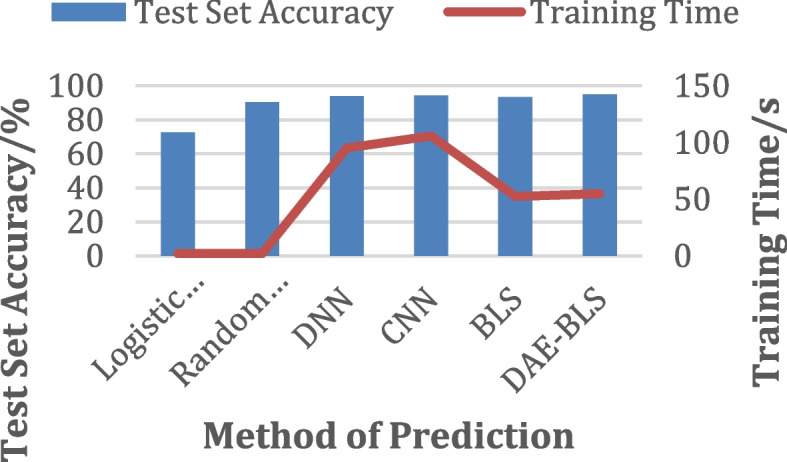
Comparison of diabetes experiment results

(2) Diabetes readmission prediction after the above pre-processing, 127,386 diabetes datasets containing 111-dimensional medical data were obtained. Compared with the heart failure database, the diabetes database has many characteristics and a large amount of data. The same algorithm was applied to the diabetes data set for experimental comparison, and the results are shown in Table 2. The comparison of results is shown in Fig. 5. The digital twin-enhanced algorithms and deep-structured neural networks usually use more parameters in order to increase accuracy. It takes a long time and a lot of technology to train most networks with a deeper structure, and it is challenging to examine the complex architecture analytically and in relation to digital twin surroundings.

**Table 2:** Diabetes prediction experiment results

Method	Training Accuracy	Test Accuracy	Training Time/s
Logistic Regression	73.21	72.46	1.98
Random Tree	91.24	90.26	2.06
Deep Neural Networks (DNNs)	95.68	93.79	95.19
Convolutional Neural Networks (CNNs)	95.02	94.22	105.76
Wide Neural Network (BLS)	94.18	93.32	52.46
DAE-BLS	95.16	94.88	54.73

Through the experimental results, it can be seen intuitively that in such a data environment with a large amount of data and complex features, the accuracy of the original BLS decreases, while the prediction model DAE-BLS proposed in this paper is not only in terms of accuracy but also in terms of It has a great advantage in training time. In contrast to alternative deep-structured neural networks, this approach remains effective. This is a result of the denoising autoencoder improving the original BLS’s capacity for feature extraction. It only requires an additional 8 s of training time, but it has resulted in a roughly 2% improvement in accuracy rate compared to the original BLS.

#### ECG data

##### Introduction to experimental datasets

This study utilises the MIT-BIH arrhythmia database [19] for conducting simulation tests. The Massachusetts Institute of Technology offers this database for the purpose of studying arrhythmia. It is one of the three globally acknowledged electrocardiogram (ECG) datasets that serve as clinical benchmarks. The MIT-BIH arrhythmia database comprises a collection of 48 dual-channel ambulatory electrocardiogram (ECG) signal records, with each record exceeding a duration of 30 min. The study participants consisted of 25 male individuals ranging in age from 32 to 89, as well as 22 female individuals ranging in age from 23 to 89. The total number of heartbeats recorded during the study was 109,500, with approximately 30% of these heartbeats classified as abnormal. The heart’s pulsations serve as a means of prognostication.

##### Abnormal heart beat test results

**Fig. 6:**
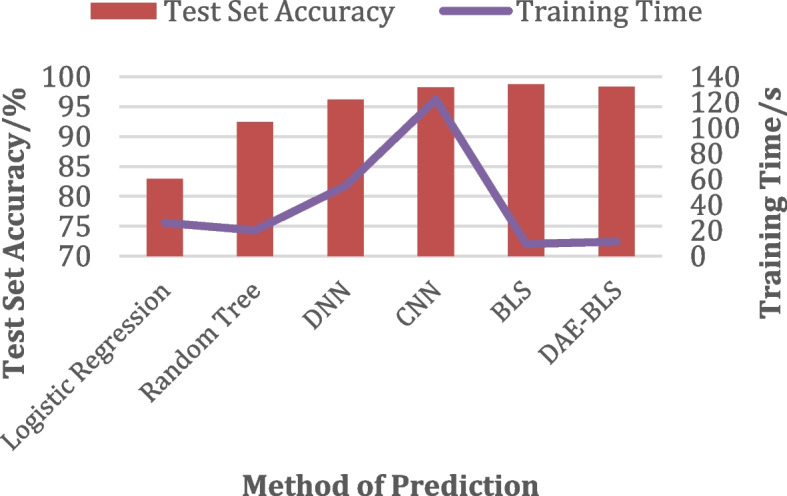
Comparison of abnormal heartbeat test results

In this paper, a series of algorithm experiments and comparisons were carried out on the abnormal heart beat data set after the above pre-processing, and the experimental results are shown in Table 3. The comparison of the results is shown in Fig. 6.

**Table 3:** Experimental results of abnormal cardiac beat prediction

Method	Training Accuracy	Test Accuracy	Training Time/s
Logistic Regression	86.26	82.91	26.00
Random Tree	96.91	92.44	20.12
Deep Neural Networks (DNNs)	98.34	96.14	54.86
Convolutional Neural Networks (CNNs)	98.19	97.72	122.31
Wide Neural Network (BLS)	98.75	97.96	9.55
DAE-BLS	98.34	98.75	11.39

It can be seen from the experimental results that the model based on the width learning system significantly improves the training speed of the model compared with other deep learning models. Deep learning requires a lot of data to function. It can be costly to train it using huge and complicated data models. Extensive machinery is also required for sophisticated mathematical calculations. Although DAE-BLS improves the accuracy of the width learning system to a certain extent, it does not improve enough. Obviously, this may be because the accuracy of the model in this dataset is already high (nearly 98%).

### Image data

In the above data set, the image data format obtained in this paper is 224 × 224 × 3, 1 148 images are the training set, and 545 images are the test set. A series of simulation experiments were carried out in this paper, and the results are shown in Table 4.

**Table 4:** Cancer CT image prediction experiment results

Method	Training Accuracy	Test Accuracy	Training Time
Logistic Regression	98.95	76.12	84.88
Random Tree	98.42	72.64	77.07
Deep Neural Networks (DNNs)	92.16	82.18	288.22
Convolutional Neural Networks (CNNs)	86.08	86.22	4699.32
Wide Neural Network (BLS)	88.77	78.10	37.24
DAE-BLS	85.52	84.75	96.18

It can be seen from the experimental results that the introduction of DAE in the width learning system has significantly improved the effect of the original BLS, and the accuracy rate has been increased by about 5%, but it also obviously takes more training time (about 30 s more ), probably because the denoising autoencoder takes more training time to process the image data. Nevertheless, the DAE-BLS model is still a fast and efficient model compared with other algorithmic models.

### Incremental learning

This paper designs experiments on the Diabetes 130-US hospitals diabetes data set used above to prove the feasibility of incremental learning used in the DAE-BLS model. First, set the initial enhancement node of the model to 1 000, and then in the initial model training If it is good, use incremental learning to add 1,000 incremental nodes for rapid reconstruction, and compare it with retraining a model. The results are shown in Table 5.

**Table 5:** Experimental results of incremental learning

Number of Enhanced Nodes/Piece	Training Set Accuracy/%	Test Set Accuracy/%	Training Time/S
1000	94.16	91.95	4.17
2000	94.35	94.25	27.15
1000→2000	94.20	94.56	9.28

It can be seen from the experimental data that it only takes 9.28 s to reconstruct the augmented nodes from 1000 to 2000 using the incremental learning algorithm, compared to 27.15 s for retraining a model with 2000 augmented nodes. The training time of nearly 17 s is saved. The experimental results show that the proposed model can achieve rapid reconstruction when the model structure needs to be changed. The introduction of DAE strengthens the feature extraction ability of BLS without destroying the superiority of its width structure. Therefore, the characteristics of high efficiency and incremental learning of BLS training are retained. Originating from the metaverse and digital twin technology, consumer health (MCH) is undergoing a paradigm change. A new era of disease prediction models has been brought about by the combination of bioinformatics and healthcare Big Data. These algorithms utilize extensive medical data to forecast illnesses before symptoms appear.

## Conclusion

Elevating the standards of healthcare and medical services has consistently been a top priority in our country. Disease prediction, in particular, represents a highly meaningful and essential endeavour. In this paper, we introduce a novel approach that leverages digital twin technology to advance the field of disease prediction. The proposed method, referred to as DAE-BLS, combines the power of denoising autoencoders with the robustness of digital twin systems, thereby enhancing the predictive capabilities of the original BLS model while retaining its efficiency in handling complex and chaotic medical data. The incorporation of digital twin technology into the disease prediction model represents a pivotal shift, allowing for more precise and agile predictions across a range of real disease datasets. However, despite the promising performance exhibited by the model on multiple datasets, certain challenges remain, necessitating further research. For instance, in the case of diabetes patient datasets, both the original BLS and the enhanced DAE-BLS exhibit less noticeable improvements in training speed compared to the heart failure dataset. This discrepancy may be attributed to factors such as dataset size and complexity, indicating the need for additional experiments with larger sample sizes and more intricate databases. Similarly, in the breast cancer dataset, DAE-BLS requires significantly more time than BLS, potentially due to the processing demands of image data by the designed DAE. To further enhance the model’s performance on image data, the structure of DAE may require refinement. In future research endeavours, we will explore more comprehensive data preprocessing techniques and leverage domain-specific medical knowledge to optimize the predictive model further. The integration of digital twin technology into the proposed method opens up new possibilities, extending its application to a broader spectrum of medical scenarios. This multidisciplinary approach, encompassing data science, medical expertise, and digital twin innovations, promises to shape the future of disease prediction and healthcare services.

## Data Availability

The datasets generated and/or analysed during the current study are available in the [UCI Machine Learning Repository] repository, [https://archive.ics.uci.edu/dataset/296/diabetes+130-us+hospitals+for+years+1999-2008].
